# Authors’ Response to Peer Reviews of “Assessment of SARC-F Sensitivity for Probable Sarcopenia Among Community-Dwelling Older Adults: Cross-Sectional Questionnaire Study”

**DOI:** 10.2196/77497

**Published:** 2025-07-25

**Authors:** David Propst, Lauren Biscardi, Tim Dornemann

**Affiliations:** 1 Department of Exercise Science, School of Health Sciences, Barton College, 200 Acc Dr W, Wilson, NC, 27893, United States, 1 252-864-6242; 2 Department of Exercise Science, School of Health Sciences, North Carolina Wesleyan College, Rocky Mount, NC, United States

**Keywords:** sarcopenia, neuromuscular, screening, community, scale, measure, questionnaires, diagnosis, gerontology, older adults, muscular

*This is the authors’ response to peer-review reports for “Assessment of SARC-F Sensitivity for Probable Sarcopenia Among Community-Dwelling Older Adults: Cross-Sectional Questionnaire Study.”*

## Round 1 Review

### Anonymous [1]

#### Major Comments

*1. Introduction: Add a discussion on current research gaps (eg, sarcopenia screening) and clearly explain how your study [2] addresses these gaps.*

**Response:** Done.

*2. Methods: Include additional clinical outcomes such as muscle function, sarcopenia-related symptoms, or quality of life, and compare how thresholds of ≥2 and ≥4 perform in relation to these outcomes.*

**Response:** We do not have additional clinical outcomes but will be sure to collect this for a follow-up study (different site, different participants).

*3. Results: Provide more detailed basic characteristics of participants and compare these between thresholds of ≥2 and ≥4, referring to Malmstrom et al [3] for guidance.*

**Response:** We do not have this information but plan to collect this for a follow-up study (different site, different participants).

*4. Discussion: Update the Discussion to integrate insights from the new results, focusing on the implications of the revised threshold for clinical practice and your limitations.*

**Response:** The Discussion was updated based on additional evaluations of the data.

### Anonymous [4]

#### Specific Comments

##### Major Comments

*1. The study looked at the association between SARC-F (strength, assistance with walking, rising from a chair, climbing stairs, and falls) and grip strength, which is not novel. Sarcopenia is poorly defined.*

**Response:** We acknowledge the existing literature on the association between SARC-F and grip strength. However, the specific novelty of our study is the validation of a lower cutoff threshold (≥2), aligning directly with The European Working Group on Sarcopenia in Older People (EWGSOP2) guidelines for earlier detection of probable sarcopenia. Additionally, our study uniquely demonstrates the practical application and clinical feasibility of this lower threshold within a routine primary care environment. Thus, we believe our study makes a novel and clinically significant contribution to the existing body of knowledge.

If the editor feels that clarification in the manuscript is necessary, then we would suggest this addition:

“Although previous studies have explored SARC-F’s relationship with grip strength, our study uniquely contributes by specifically validating the clinical practicality and efficacy of a lower threshold (≥2) within a primary care setting. This approach directly addresses the EWGSOP2’s recommended strategy for early detection.”

*2. The sample size needed to be more adequate, and only 11% of the subjects had lower grip strength.*

**Response:** We acknowledge the reviewer’s concern regarding our sample size and low prevalence of probable sarcopenia. Despite this limitation, our analyses showed robust statistical power (99.5%), validating the utility of our findings for our clinical setting. We have explicitly recommended future larger, multicenter studies within our manuscript’s Limitations section to confirm the generalizability and validity of our results. Thus, we believe no manuscript changes are necessary.

*3. It is acceptable if it is used for estimation or prediction, such as death, but an area under the curve (AUC) of 0.77 may be too low as an index for diagnosis and discrimination.*

**Response:** We appreciate the reviewer’s concern about the AUC value. We emphasize that our intention was to evaluate SARC-F as an initial screening tool—not a definitive diagnostic test. An AUC of 0.77 is appropriate and aligns with values reported in comparable sarcopenia screening studies. To clarify, we have emphasized in our manuscript that the reported AUC supports the feasibility and clinical relevance of the SARC-F threshold as an initial screening tool.

If the editor feels that clarification in the manuscript is necessary, then we would suggest this addition:

“Our observed AUC of 0.77 aligns well with other validated sarcopenia screening studies (eg, [5]). It is essential to recognize that initial screening tools like SARC-F are not intended for definitive diagnostic accuracy but rather for effectively identifying patients who should undergo further evaluation. Thus, this moderate AUC value supports the feasibility and clinical utility of the SARC-F at a threshold of ≥2.”

*4. The Methods describe too few details, and Table 1 provides too little background information.*

**Response:** We thank the reviewer for suggesting more detailed participant characteristics. However, due to data access limitations, we have no additional comorbidities or information available.

We have given a detailed suggested text for the Methods section to include more detailed descriptions of all variables collected, how they were measured, and a clearer explanation of the statistical analyses used—particularly the rationale for receiver operating characteristic (ROC) analysis and effect size reporting.

If the editor feels that clarification in the manuscript is necessary, then we would suggest this addition:

“Data Collection

“Data were collected from de-identified clinical records and included age, gender, BMI, SARC-F scores, and grip strength. SARC-F was administered during routine visits, and grip strength was measured using a calibrated digital dynamometer following a standardized protocol (see Grip Strength subsection).

“Statistical Analysis

“Normality was assessed using the Kolmogorov-Smirnov test and histograms. Between-group comparisons were conducted using independent t-tests for normally distributed data and Mann-Whitney U tests for non-parametric data. ROC analysis was conducted to assess the ability of the SARC-F score to discriminate between individuals with and without probable sarcopenia (defined by EWGSOP2 grip strength thresholds). The area under the curve (AUC) was calculated, and optimal SARC-F thresholds were identified. Sensitivity, specificity, predictive values, and accuracy were calculated across cutoffs. Effect sizes (Cohen d or r) were reported to assess clinical relevance of differences. A post-hoc power analysis of the ROC confirmed 99.5% power.”

*5. Ultimately, the conclusions that can be drawn from the results should be revised.*

**Response:** We thank the reviewer for the important reminder to align the study’s conclusions with its objectives and data. We have revised the conclusion to clearly reflect the feasibility and screening utility of the SARC-F at a lower threshold while avoiding overstatement regarding diagnostic application.

We do agree with this and would suggest this text:

“Conclusion

“This study supports the use of a lower SARC-F threshold (≥2) as a feasible and effective screening tool to identify older adults at risk for probable sarcopenia in primary care. The threshold improves sensitivity while maintaining acceptable specificity, enhancing early detection. These findings are particularly relevant for busy or resource-limited clinical settings where quick, non-invasive screening methods are needed. While SARC-F should not be used as a diagnostic tool alone, a lower cutoff can reliably prompt further assessment of muscle strength and timely intervention, aligning with EWGSOP2 recommendations for early clinical action.”

I would like to thank the reviewers and editors for the time that was spent on my project. I do see the comments as an attempt to make my work on this project better and to improve any future work.

## Round 2 Review

### Anonymous [1]

*Thank you for your revisions. I understand that due to the lack of relevant data, you were unable to expand your data analysis. I am pleased to see the addition of Tables 3 and 4 for the subgroup analysis; however, these two tables could be combined. Additionally, you may consider placing the ROC curves from Figures 1 and 2 into a single figure. Using software like MedCalc or SPSS to compare the areas under the different ROC curves would add more depth to the Results section.*

**Response:** Combine Tables 3 and 4, statistically compare AUCs, and merge ROC curves. Tables merged into Table 3 (page 8). ROC curves combined into Figure 2, and DeLong test added (Results, page 7,  lines 205‐210; *P*=.98). Improved figure resolution. Uploaded 600 DPI TIFFs for Figures 1 and 2.

### Anonymous [4]

#### Specific Comments

##### Major Comments

*To begin with, SARC-F is a screening indicator for sarcopenia, not for probable sarcopenia (decreased grip strength). If you try to find a cutoff for probable sarcopenia, which is a prestage of sarcopenia, the cutoff value will inevitably be smaller than the cutoff value used to determine sarcopenia. With that in mind, how do you explain the significance of this paper? Please argue the need to screen for decreased grip strength with a cutoff of 2 points rather than screening for sarcopenia with a cutoff of 4 points.*

**Response:** Thank you for highlighting this point. We have clearly acknowledged the distinction between sarcopenia and probable sarcopenia as per EWGSOP2 guidelines. Our manuscript emphasizes that identifying probable sarcopenia at an earlier stage facilitates earlier clinical intervention, aligning with EWGSOP2 recommendations. Thus, we believe no manuscript changes are necessary.

*In addition, the cutoff of 2 points on a questionnaire consisting of five items with a range of 0‐12 points is an extremely low value. The question that arises here is whether there is any point in using this questionnaire in the first place. The authors will first need to show which of the lower-level items contribute strongly to the prediction of grip strength decline as a sensitivity analysis. Then, they should also mention whether the SARC-F should be used as a questionnaire indicator or whether it would be better to use the lower-level items as a new screening indicator.*

**Response:** Thank you for highlighting this important aspect. We agree that emphasizing the clinical utility and practical feasibility of adopting the lower SARC-F threshold (≥2) is essential. We have clarified in our Discussion that this lower threshold promotes earlier detection, supports timely intervention, and easily integrates into routine clinical workflows, especially in resource-limited settings. We have provided additional text in our Discussion to further underscore these points.

If the editor feels that clarification in the manuscript is necessary, then we would suggest this addition:

“Clinically, the adoption of a SARC-F threshold of ≥2 enhances early detection and timely intervention, improving patient outcomes and reducing progression to advanced sarcopenia. Our findings support the feasibility of using this lower threshold routinely in primary care, particularly due to the minimal additional time or resources required for implementation.”

##### Minor Comments

*Information on ethical matters is lacking.*

1. *Is there an ethics approval number?*
2. *It is said that informed consent was not required, but how was information disclosed to the research subjects regarding your research? Was an opt-out notice posted?*
3. *How was the opportunity for the subjects to decline participation in your research provided?*

*It says “regularly scheduled physician visits,” but is this study a single or multicenter study?*

*What is the reason for the subjects’ physician visits? Are the subjects suffering from some disease? If so, the disease information may be an important confounding factor in this study, so please clearly state the results and show them in Table 1.*

**Response:** We thank the reviewer for emphasizing the importance of clearly documenting ethical procedures. I have uploaded the institutional review board letter to the manuscript account. The SARC-F questionnaire and grip strength testing were performed as part of the patient’s routine physical exam along with vital signs and weight. Patients are able to refuse any screening that they do not wish to have completed.

*Please show the inclusion and exclusion criteria for the subjects.*

**Response:** We appreciate this suggestion. We have clarified and explicitly detailed the inclusion and exclusion criteria in the Methods section to enhance transparency and facilitate a better understanding of our study population and the generalizability of our findings.

We do agree with this and would suggest this text:

“Participants included community-dwelling older adults aged 65 years and older, attending routine primary care appointments, and capable of performing grip strength testing and completing the SARC-F questionnaire. Individuals were excluded if they were unable or unwilling to complete the grip strength assessment due to acute medical conditions, recent injuries, significant arthritis, neurological conditions, or substantial cognitive impairment interfering with questionnaire completion. These criteria were designed to reflect realistic primary care screening practices, ensuring patient safety, test accuracy, and data validity.”

*Who measured grip strength, where, and in what position?*

**Response:** We appreciate the reviewer’s request for additional measurement details. We have clarified our grip strength measurement procedures in the Methods section, including information about personnel, equipment, and standardized measurement protocols to ensure reproducibility and consistency.

We do agree that clarification would be beneficial and would suggest this text:

“Grip strength was assessed in private exam rooms by the same staff member for all assessments. Participants were seated comfortably with elbows flexed at 90°, forearm and wrist in neutral positions, and feet flat on the floor. Using a digital dynamometer (Sutekus Digital), participants completed three maximal grip attempts lasting 3‐5 seconds each, with approximately 30‐60 seconds of rest between trials. The highest recorded grip strength value from the dominant hand was utilized for analysis.”

*In the Statistical Analysis section, it says “visual histograms,” but they are not shown in the Results. Please show them. In particular, it would be desirable for the histogram of the SARC-F score to be free from extreme bias when conducting the analysis. Please show the histogram for each sex and show that the sampling is appropriate for verifying the value conducted in this study.*

**Response:** We appreciate this suggestion. We have added histograms of SARC-F score distributions by sex to visually demonstrate the nonnormal distribution. These figures support our use of nonparametric methods and enhance the transparency of our statistical approach.

The histogram is being shared here but is also being uploaded.

Suggested caption: “Figure X. Distribution of SARC-F Scores by Sex. Histograms showing the distribution of SARC-F scores among male (left) and female (right) participants. Scores are clustered at the lower end of the scale in both groups but display greater dispersion and right skew among females. These distributions support the use of non-parametric statistical methods for between-group comparisons.”

*Before validating the cutoff value of the SARC-F based on grip strength, it’s crucial to establish a robust relationship between grip strength and the SARC-F. This can be achieved through multiple regression analysis, with grip strength as the dependent variable, the SARC-F as the explanatory variable, and other factors as adjustment factors. This step is essential to ensure the validity of the research.*

**Response:** Thank you for this valuable suggestion. Regression analysis was beyond our original study’s scope, but we agree this would significantly strengthen understanding of the predictive relationship between SARC-F and grip strength. Therefore, we have not suggested any changes to our manuscript.

*The factors that may confound the relationship between SARC-F and grip strength have yet to be sufficiently demonstrated. For example, what about cognitive function and physical activity?*

**Response:** We appreciate the reviewer’s suggestion regarding confounding variables. While cognitive function and physical activity were not included in our original analysis, we acknowledge their importance and have explicitly recommended in our Limitations section that future research should incorporate these factors to better clarify their potential influence on the relationship between SARC-F scores and grip strength.

We do agree that clarification would be beneficial and would suggest this text:

“Our study did not include potential confounders such as cognitive function or physical activity levels, which may influence SARC-F responses and grip strength performance. Future research should incorporate these variables to enhance our understanding of their potential mediating or moderating effects on sarcopenia screening outcomes.”

*The male’s grip strength of 36.3 kg is extremely strong for a subject who should be selected for probable sarcopenia. There is a high possibility of selection bias. Please clearly state in the Discussion how you interpret this point.*

*As mentioned above, much important information needs to be included, and even though there are limitations from the research planning stage, they should be mentioned in the Discussion.*

*If you do not present the information mentioned above, please clearly state the limitations of the research in the Discussion section, and also explain why you still think the research results are meaningful and why it is necessary to make the results of this research public.*

**Response:** Thank you for this recommendation. We have expanded the Limitations section (see suggested text) to include potential sources of bias and the cross-sectional design limitations, and we have justified the continued clinical value of our findings in light of these constraints.

If the editor feels that clarification in the manuscript is necessary, then we would suggest this addition:

“This study has several limitations. First, its cross-sectional design does not allow for conclusions about causality or changes in muscle strength over time. Second, because participants were community-dwelling older adults attending routine care visits, there is a potential for selection bias, as individuals with significant frailty or cognitive impairment may have been excluded. Third, reliance on self-reported SARC-F data may introduce recall or reporting bias. Fourth, while age, sex, and BMI were recorded, other potentially influential variables such as comorbidities, physical activity levels, and cognitive function were not systematically assessed. These factors may act as confounders in the relationship between SARC-F and grip strength. Despite these limitations, the study’s high statistical power and real-world clinical design provide strong support for the feasibility of a lower SARC-F threshold in routine screening.”

## Abbreviations

AUC: area under the curve
EWGSOP2: The European Working Group on Sarcopenia in Older People
ROC: receiver operating characteristic
SARC-F: strength, assistance with walking, rising from a chair, climbing stairs, and falls
